# Multi-purpose SLM-light-sheet microscope

**DOI:** 10.1364/BOE.9.005419

**Published:** 2018-10-12

**Authors:** Chiara Garbellotto, Jonathan M. Taylor

**Affiliations:** University of Glasgow, University Avenue, Glasgow, G12 8QQ, UK

## Abstract

By integrating a phase-only Spatial Light Modulator (SLM) into the illumination arm of a cylindrical-lens-based Selective Plane Illumination Microscope (SPIM), we have created a versatile system able to deliver high quality images by operating in a wide variety of different imaging modalities. When placed in a Fourier plane, the SLM permits modulation of the microscope’s light-sheet to implement imaging techniques such as structured illumination, tiling, pivoting, autofocusing and pencil beam scanning. Previous publications on dedicated microscope setups have shown how these techniques can deliver improved image quality by rejecting out-of-focus light (structured illumination and pencil beam scanning), reducing shadowing (light-sheet pivoting), and obtaining a more uniform illumination by moving the highest-resolution region of the light-sheet across the imaging Field of View (tiling). Our SLM-SPIM configuration is easy to build and use, and has been designed to allow all of these techniques to be employed on an easily reconfigurable optical setup, compatible with the OpenSPIM design. It offers the possibility to choose between three different light-sheets, in thickness and height, which can be selected according to the characteristics of the sample and the imaging technique to be applied. We demonstrate the flexibility and performance of the system with results obtained by applying a variety of different imaging techniques on samples of fluorescent beads, zebrafish embryos, and optically cleared whole mouse brain samples. Thus our approach allows easy implementation of advanced imaging techniques while retaining the simplicity of a cylindrical-lens-based light-sheet microscope.

## 1. Introduction

Selective Plane Illumination Microscopy (SPIM) [1] is becoming increasingly popular for live fluorescence imaging in developmental biology. Compared to confocal microscopy, which also performs optical sectioning, the use of a static light-sheet to illuminate only the imaging focal plane means that SPIM offers important advantages including rapid snapshot acquisition and reduced photobleaching of the sample [2]. Snapshot acquisition is particularly important for capturing the 3D structure of rapidly changing organisms or scenes. Photobleaching is minimized by the confinement of the illumination light to the single plane which is currently being imaged, thus ensuring that each fluorophore is excited only for the time required to expose that particular plane of the sample. Despite these attributes making SPIM particularly well-suited for *in vivo* imaging, a classical SPIM implementation suffers from a number of issues including:

- Shadow artefacts: parts of the sample will absorb or scatter the side-launched light-sheet, generating dark stripes behind them, elongated parallel to the illumination direction.
- Scattering: when illuminating a plane inside the sample, light emitted by the excited fluorophores has to travel through sample tissue in order to be collected by the imaging objective, which means it inevitably undergoes some scattering on the imaging path. This results in undesirable out-of-focus background in the images, leading to reduced image contrast. Tissue scattering also affects the propagation of the light-sheet itself, resulting in even more out-of-focus light, in this case coming from out-of-focus fluorophores excited by the scattered light-sheet.
- Limited field of view: even in the absence of scattering, the illumination delivered by a Gaussian light-sheet is not uniform in thickness across the image Field of View (FoV). A Gaussian beam generates a light-sheet with a certain waist size (thickness of the light-sheet at its focus) and extent (Rayleigh length). This shape of the light-sheet results in an uneven illumination across the image FoV, with better optical sectioning around the beam waist, where the light-sheet is at its thinnest, and poorer optical sectioning (more out-of-focus excitation) at the sides of the image, generated by the thicker parts of the sheet.

A variety of modifications to SPIM light-sheet microscopy have been proposed to tackle some of these issues, and similarly for the closely-related technique of DSLM light-sheet microscopy (Digital Scanned Laser Light-sheet Fluorescence Microscopy [3]), where a light-sheet is synthesized by rapid scanning of a focused 2D Gaussian beam. Shadows can be reduced by illuminating the sample from multiple directions [4, 5], and using Bessel [6–8] or Airy [9] beams instead of Gaussian beams permits a more uniform illumination across a larger FoV. One way to reduce the effects of scattering from tissue surrounding the imaged plane is to use a DSLM configuration (where a synthetic light-sheet is formed from a scanned Gaussian beam) in conjunction with a rolling confocal slit on the detection camera to reject scattered light [10]. Methods based on structured illumination, such as HiLo [11] and the method proposed in [12] which we refer to as the 3-phase method, can also help enhance image contrast by reducing the out-of-focus contribution. The technique known as tiling can be used to extend the limited FoV over which high-quality depth sectioning can be achieved using a simple Gaussian beam [13]. In this case each plane in the sample is imaged multiple times, each time with the light-sheet focused at a different lateral position in the FoV. The final image of the plane is then created by stitching together adjacent vertical stripes taken from the different images, each stripe containing only the part of the image generated by the thinnest part of the light-sheet.

The aim of the present paper is to present a single SPIM system that uses a spatial light modulator (SLM) to create a simple and flexible platform for performing the aforementioned advanced light-sheet microscopy techniques, as well as permitting automatic adaptive light-sheet positioning for best focus (autofocusing). Our setup consists of a basic SPIM microscope (illumination arm with cylindrical lens, imaging chamber with sample holder and motorized translation stage for z-scanning, imaging arm) with the addition of a phase-modulating SLM in the illumination arm. With the SLM conjugated to a Fourier plane in the optical path of the illumination beam (SLM illuminated by a collimated light beam), we demonstrate that it is possible to modulate and move the resultant light-sheet to implement a wide range of imaging techniques. While SLMs have previously been used for certain specific purposes in light-sheet illumination [9, 14–16], we will show that a single microscope design is well-suited for applying a wide variety of different optical techniques on the same imaging platform.

This work is organized as follows. In the next section we present the optical design of our SLM-SPIM microscope, discuss the motivation behind our design choices and the general advantages and limitations offered by such system. We conclude the section with details about the different samples used in our experiments and the way they were mounted for imaging. In Section 3 we introduce some of the imaging techniques that can be performed with our system, and include experimental results obtained imaging fluorescent beads in agarose, Zebrafish embryos, and cleared mouse brain samples. In Section 3.3 and 3.4 we also propose and discuss new reconstruction methods based on the maximum intensity projection operation. All the raw data and the MATLAB codes used to produce figures, plots and calculations presented in Section 3 can be found in the data repository [17].

## 2. SLM-SPIM microscope

SLMs are versatile pixelated phase (or amplitude) modulation devices that have already found many applications in the field of optical microscopy [18], where they have been used to control the sample illumination [19] or as a Fourier mask in the imaging path [20, 21]. SLMs have previously been described by a few authors in SPIM systems, both in the illumination and in the imaging path (for example to deliver structured illumination [16] or to correct for aberrations [14]). Recent work in parallel with our own has presented the SSPIM (Structured SPIM [22]), a DSLM microscope with an incorporated SLM which offers the option to shape the beam profile (Gaussian, Airy, Bessel, Lattice) as well as performing tiling and structured illumination. In contrast, our work showcases the use of a programmable SLM device in a more classical scan-free, cylindrical-lens-based SPIM such as that used for the OpenSPIM design [23].

**Fig. 1:**
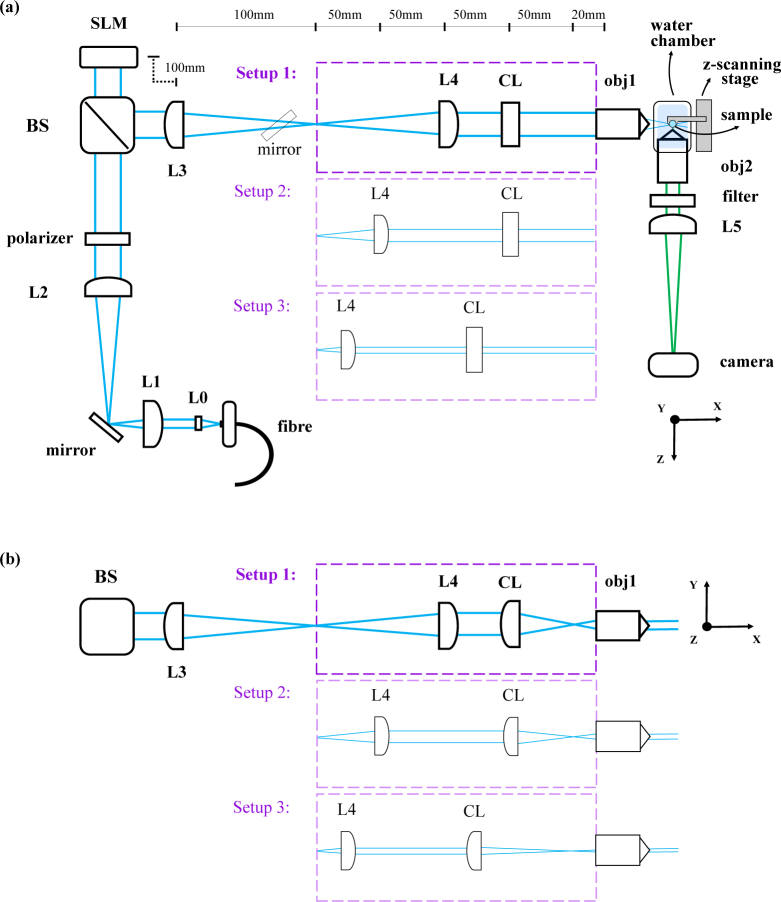
Optical scheme of our SLM-SPIM, with a top view of the system in (a) and a side view of its launching arm in (b) (see Table 1 for details of the individual components). Changing the position of the last two lenses before the illumination objective allows us to switch between three different setups, yielding different sheet heights and thicknesses, and changing the conjugation of the SLM with the sample plane. (a) View of the SLM-SPIM from above. The cylindrical lens has no optical power in this plane. A mirror placed before the SLM (bottom right corner) permits adjustment of the vertical position of the light-sheet in the sample plane. A second mirror can easily be inserted after L3 and used to redirect the laser beam to the side (upwards in this figure), onto a second illumination arm, (not included in this scheme) ending in the glycerol chamber. The glycerol illumination arm consists of (see Table 1 for details): L3 (shared with the water-imaging system) ← 160mm→ CL2 ←40mm→ L6 ←100mm → obj3 → glycerol chamber. The glycerol imaging arm is mounted vertically above the glycerol chamber and is composed of a glycerol dipping objective (obj4), a tube lens (L7) and the same camera used for the images in water. (b) Side view of the final part of the SLM-SPIM illumination arm, with three different possible configurations.

In our SPIM we use a reflective phase-only liquid crystal SLM, placed in a Fourier plane in the optical path of the illumination beam. The optical setup is illustrated in Fig. 1

, while details of the components and devices used are summarized in Table 1

**Table 1:** List of components used (with reference to Fig. 1).

**Lenses**	**L0:** achromatic doublet (Thorlabs, AC064-013-A-ML), *f* = 13 mm; **L1:** plano-convex, *f* = 35 mm; **L2:** plano-convex, *f* = 100 mm; **L3:** plano-convex, *f* = 100 mm; **L4:** plano-convex, *f* = 100 mm for setup1, *f* = 50 mm for setup 2 and *f* = 25.4 mm for setup 3; **CL:** cylindrical lens, *f* = 50 mm for setup 1 and 2, *f* = 80 mm for setup 3; **L5:** plano-convex, *f* = 100 mm;
**Other optical elements**	**BS:** beam splitter; **polarizer**: linear polarizer;**filter:** Green Fluorescent Protein (GFP) filter (central wavelength 525 nm); **obj1:** 10× Nikon Plan Fluorite Imaging Objective, 0.3 NA, 16 mm WD (Working Distance); **obj2:** 40× Nikon CFI APO NIR Objective, 0.80 NA, 3.5mm WD;
**SLM**	Hamamatsu LCOS-SLM (Liquid Crystal on Silicon Spatial Light Modulator) serie X13138;
**SLM head**	**pixels:** 1272 × 1024; **pixel size:** 12.5 *µ*m; **effective area size:** 15.9 mm × 12.8 mm; **fill factor:** 96 %;
**SLM controller**	**input signal:** DVI-D; **DVI signal format (pixels):** 1280 × 1024; **input signal levels:** 256; **DVI used frame rate:** 60Hz;
**Laser**	OBIS coherent laser; **wavelength:** 488 nm;
**Camera**	XIMEA MD028xU-SY; **sensor active area:** 8.8 mm × 6.6 mm; **resolution:** 1934 × 1456, 2.8 Mp; **pixel size:** 4.54 *µ*m; **frame rate:** 56.9 fps; **dynamic range:** 71.1 dB;
**Glycerol system**	Illumination arm, after the SLM, L3 and the pop-in mirror: **CL2:** cylindrical lens, *f* = 60 mm; **L6:** plano-convex, *f* = 100 mm; **obj3:** 5× ZEISS EC Plan-Neofluar Objective, 0.16 NA, 18.5 mm WD. Imaging arm: **L7:** plano-convex, *f* = 150 mm; **obj4:** 20× ZEISS Clr Plan-Neofluar Objective, 1.0 NA, 5.6 mm WD;

. The laser beam coming out of the optical fiber is collimated, expanded (to fill the size of the SLM active surface), and directed onto the SLM, which modulates the phase of the beam’s wavefronts. Two lenses and a cylindrical lens are then used to create the light-sheet and conjugate the plane of the SLM to the back focal plane of the illumination objective. This conjugation means that what the objective focuses (in one direction) on the sample is the far field diffraction pattern corresponding to the phase-shift pattern displayed on the SLM, i.e. its Fourier transform.

As is standard for SLMs, the phase-shift patterns referred to in this article were generated through the following steps. First, a continuous phase function Φ(*x, y*) is defined. This function is then discretized into Φ(*x_i_, y_i_*), where (*x_i_, y_i_*) for *i* = 1 : *N* are the *N* pixels of the SLM, so that each value Φ(*x_i_, y_i_*) contains the phase-shift which will be applied to the incoming wavefront by the *i*-th pixel of the SLM. The values of Φ are then wrapped at 2*π*, and Φ is saved as a 8-bit image and displayed on the SLM active area. In the process of converting the phase function into an image, phase-shift values ranging from 0 to 2*π* are converted into numbers from 0 to 255 (taking into account the calibration of the specific SLM model used - for our model a value of 138 gives a phase shift of 2*π* at 488 nm).

A second system optimized for imaging cleared mouse organs (glycerol/CLARITY) shares common laser launch optics and SLM with our water-immersion system, but has a separate final part of the illumination arm (last spherical lens, cylindrical lens and launching objective), glycerol chamber, and a vertically mounted imaging arm (components included in Table 1). The patterns displayed on the SLM can easily be rotated and customized for either one of the systems, permitting us to perform the different imaging techniques on both water- and glycerol-immersed samples. In both cases the sample is mounted on a motorized translation stage, and 3D imaging is performed by scanning the sample through the light-sheet.

As illustrated in Fig. 1, our water-immersion system also allows easy switching between three different optical configurations on the laser launch path. Each of these interchangeable modules has a different positioning of the cylindrical lens and final spherical lens. This allows us to choose between three light-sheets of different thickness and height, as well as offering different conjugations between the SLM and the objective lens (discussed in detail below). Experimental profiling of the light-sheet (using a small fluorescent bead translated through the light-sheet) has confirmed a FWHM (at sheet waist) of around 2 *µ*m for setup 1, 3 *µ*m for setup 2 and 5 *µ*m for setup 3. The sheet height is ~4 mm for setup 1, ∼2 mm for setup 2, and ∼0.6 mm for setup 3. On the imaging side, the use of a 40× Nikon objective with a 100 mm tube lens gives us an imaging FoV of ∼440 *µ*m × 330 *µ*m, with a lateral resolution at *λ* = 0.525 *µ*m of 0.4 *µ*m (calculated as 0.6×*λ* / NA). In the glycerol imaging arm, the combination of the 20× ZEISS objective and the 150 mm tube lens gives a FoV of ∼490 *µ*m × 370 *µ*m and a lateral resolution at *λ* = 0.525 *µ*m of 0.3 *µ*m.

When performed on our SLM-SPIM microscope, all the discussed imaging techniques involve the post-acquisition combination of more than one image of the same plane. With our work, we would in fact like to show how multiple-image acquisition can open up new possibilities for improved image reconstruction (see discussion in Section 3.3). The speed at which the final image of a single plane can be produced therefore depends on the number of images required by the specific imaging technique, and the rate at which images can be acquired. If effort were in future to be focused on optimising our platform for imaging speed, then a key rate-limiting factor would be the SLM maximum update rate, which for our SLM model is 60 Hz.

### 2.1. Design considerations

In the light-sheet launch path, it is necessary to place the cylindrical lens after the SLM. Otherwise, if the SLM is appropriately conjugated to a pupil or image plane, a line focus will be formed on the SLM and no meaningful phase control would be available along one axis. However, it follows from this choice that it is not possible for the SLM to be simultaneously conjugate to the pupil plane in both the horizontal and vertical axes simultaneously (and similarly, neither can it be simultaneously conjugate to the object plane in both axes). These considerations mean that care is required in designing the light-sheet launch optics to ensure that the SLM provides the necessary degrees of freedom to manipulate the light-sheet as required for an experiment.

The three interchangeable lens modules mentioned above offer different conjugations between the plane of the SLM and the focal plane of the illumination objective, each of which is optimal for different families of applied beam shaping techniques. In a top-down view of our system design (Fig. 1a), it can be seen that setup 1 conjugates the SLM with the back focal plane of the illumination objective: the two lenses L3 and L4 are separated by the sum of their focal lengths, the SLM is at *f*_3_ from L3 and the back focal plane of the objective is at *f*_4_ from L4.

However, the optical power of the cylindrical lens means that, when viewing the system from the side (Fig. 1b), the SLM is not conjugate to either a pupil or image plane. In contrast, setup 2 gives the opposite situation and, in its side view, it conjugates the plane of the SLM with the focal plane of the launching objective: L4 and the cylindrical lens are separated by the sum of their focal lengths and the back focal plane of the objective is at *f_cl_* from the cylindrical lens. Setup 3 gives a conjugation similar to the one of setup 2, but was designed to give a good compromise between a perfect conjugation (achievable with the use of a cylindrical lens with *f_cl_* = 75 mm, instead of the *f_cl_* = 80 mm we decided to use for this setup) and the combination of high flexibility in tilting the light-sheet and a high demagnification of the incoming beam (both of which are achieved using a longer *f_cl_*), resulting in a light-sheet with thicker waist and longer Rayleigh length (see Section 3.3 for a more detailed discussion of the reasons behind these choices).

Most of the imaging techniques we mention in this article successfully improve image quality when performed using any of the three setups of our system. Nonetheless, because of the different sheet height, sheet thickness and SLM conjugation they provide, the best results are achieved by selecting, for each imaging technique, the most appropriate of three setups. Let us for example consider the light-sheet thickness. Setup 1 gives the thinnest light-sheet at beam waist, and is therefore the best one to use for experiments such as the light-sheet tiling ones (Section 3.2), where the only part of the light-sheet which is actually used to create the final image is its central, thin waist. Setup 3 gives the thickest light-sheet, which on the other hand also means a more even illumination across the FoV (longer Rayleigh length). This makes it a good choice for experiments such as the pencil beam scanning in Section 3.4, where the light-sheet illumination is substituted with a vertically scanned focused beam. The use of a thicker but more uniform beam also helps reduce the time needed to generate a homogeneously illuminated image of the entire FoV. As specified in Section 3.4, the results we chose to include for the pencil beam scanning technique (Fig. 6) were obtained using the glycerol setup, but for the above reasons we used (and would suggest using) setup 3 when performing this technique on the water system. Finally, in terms of sheet thickness and Rayleigh length, setup 2 offers a compromise between setup 1 and 3.

The choice of what setup to use should also depend on the characteristics of the sample to be imaged, and not only on the specific imaging technique to be implemented. For the experiments which we could perform with more than one of the three setups, we chose to use setup 1, which is the one that we find gives the best light-sheet for imaging fluorescent beads and Zebrafish embryos, with a vertically uniform illumination across the used FoV and a 2 *µ*m thick light-sheet waist. In Section 3.3 (shadow suppression experiments) we give an example of a situation in which the type of sample strongly influences the setup choice, further demonstrating the advantages of working with an easily reconfigurable system.

In our experiments, we use the SLM in a refractive mode (applying blazed gratings). This means that most of the light modulated by the SLM is concentrated in the first diffracted order, but some power is always lost in higher orders and in the 0th order (containing the specularly-reflected light that is not modulated by the SLM). In order to eliminate the 0th order we apply a further, constant, horizontal phase ramp (phase tilt) to the SLM, to displace the desired first order from the 0th order. The SLM is then physically tilted (and fixed) such that the first order is brought back to the optical axis, and the 0th order is instead left to the side. A vertical (along *y*) slit mask placed in the first focal plane after the SLM (focal plane of L3 in Fig. 1) is finally used to block this 0th (and higher) orders. We apply this approach to all the imaging techniques discussed in the following sections, with a small difference only for the autofocusing technique described in Section 3.5, where the masking procedure is conceptually the same but rotated of 90 degrees: the additional phase ramp is added vertically to the SLM, the slit mask is elongated along *z* and the SLM is tilted vertically. When we apply different phase patterns to the SLM (to perform the different imaging techniques), the trajectory and structure of the first order beam is altered slightly, but the addition of the constant phase ramp combined with the tilt of the SLM makes sure it always passes through the mask. Both the specific pattern and the constant phase ramp added to it have an influence on the diffraction efficiency of the SLM, defined as the ratio of the first order intensity to the 0th order intensity when no pattern is applied to the SLM. For a phase ramp with a spatial frequency of 20 lines per millimetre (4 SLM pixels), we calculated a diffraction efficiency (at 488 nm) of ∼60 %, in accordance with the manufacturer’s specifications for our SLM model. In case of limited laser power availability, the overall diffraction efficiency could be increased by applying a lower-spatial-frequency tilt to the patterns, at the cost of requiring greater care in the masking procedure, since the first diffraction order would then be focused closer to the 0th order.

### 2.2. Samples

For our experiments with the water objective, the samples were mounted in a length of FEP tubing (Fluorinated Ethylene Propylene, 1.3 mm ID × 1.6mm OD, Adtech Polymer Engineering Ltd) attached to the end of a syringe. For calibration and alignment purposes, and for the tiling experiments, the FEP tube was filled with beads (polystyrene beads, 0.2 *µ*m Dragon Green, Bangs Laboratories Inc) in a 1.5 % low melting point agarose solution (Agarose, High-EEO/Protein Electrophoresis Grade, Fisher Scientific). When imaging ex-vivo Zebrafish embryos, the fish was placed in a FEP tube filled with system water. For our experiments we used the Zebrafish nacre mutant (reduced pigment), with the cardiac muscle labelled with green fluorescent protein (*myl7:eGFP*). The fish specimen was preserved in formalin (10% Formalin solution, neutral buffered, Sigma-Aldrich). For the experiments on cleared mouse samples, we used a mouse brain prepared with the CLARITY method [24]. The sample was placed in a quartz cuvette (UV fused quartz glass, Thorlabs part CV10Q3500F) filled with 85% glycerol, which was sealed while excluding any air bubble from inside. The cuvette was then immersed in the chamber filled with 85% glycerol, and held horizontally underneath the dipping imaging objective.

## 3. Results and analysis

### 3.1. Structured Illumination

**Fig. 2:**
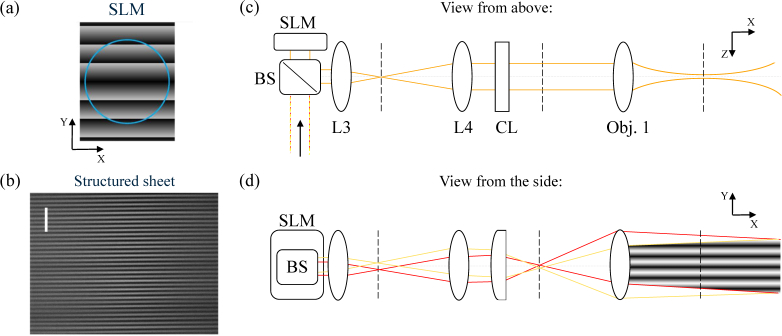
(a) Pattern displayed on the active area of the SLM to create a sinusoidally modulated light-sheet; the blue circle indicates the footprint of the collimated input beam. (b) Experimental image of a modulated light-sheet obtained with our method, imaged in aqueous Fluorescein dye diluted in water, revealing an interference pattern with a period of ∼10*µ*m (white to white); scale bar: 50 *µ*m. (c) Schematic showing the optical path (distances and sizes not to scale) of the light reflected off the SLM. The two beams follow the same optical path when viewed in the *xz* plane, forming two light-sheets on the same plane. (d) In the *xy* plane, the two sheets propagate at different angles, generating an interference pattern in the sample plane.

Two mutually-coherent light-sheets that propagate in the same plane (*x y* plane), but at a different angle with respect to the optical axis of the illumination arm, will interfere in the sample plane and generate a light-sheet with sinusoidal modulation along *y* (Fig. 2b

). This type of intensity-modulated light-sheet can be used to perform two different structured illumination techniques: HiLo [11, 25], and the 3-phase method [26, 27]. In the 3-phase method, three images are taken by translating the same sinusoidal light-sheet by precise spatial phase shifts of: 0, 2/3*π* and 4/3*π*. The three images I_1,2,3_ are then combined using the following formula:

$$I=\frac{\sqrt{2}}{3}{\left[({I_{1}-I_{2})}^{2}+{\left(I_{1}-I_{3}\right)}^{2}+{\left(I_{2}-I_{3}\right)}^{2}\right]}^{1/2},$$ (1)

to yield a final image I with reduced out-of-focus background and hence enhanced contrast. This 3-phase technique has been implemented on a digitally-scanned light-sheet microscope by fast time-modulation of the scanned beam [28], and in 2007 on a SPIM microscope using a grid-projection approach [12]. HiLo imaging has also been implemented previously on a digitally-scanned light-sheet microscope; it requires only two raw images, one acquired with a normal, uniform light-sheet and one with a modulated light-sheet [11].

**Fig. 3:**
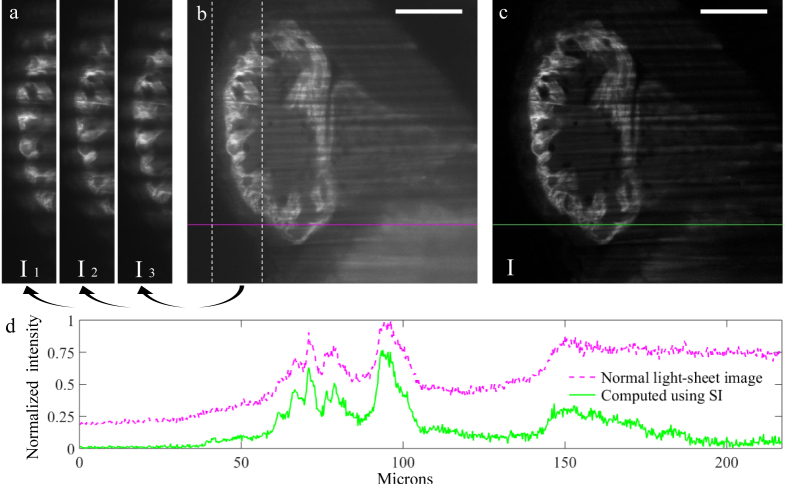
3-phase structured illumination performed using setup 1. (a) Cropped views of the three images, *I*_1,2,3_, taken with a modulated light-sheet (period = 20 *µ*m on the sample plane). The sinusoidal pattern for the second and third images is shifted by a phase of $\frac{2}{3}\pi$ and $\frac{4}{3}\pi$ with respect to the first image. (b) Image acquired with a normal, non-modulated light-sheet. (c) Image obtained by combining *I*_1_, *I*_2_ and *I*_3_ using Equation 1. (d) Intensity profile along the same row in images (b) and (c), to visualize the achieved background reduction and improved image contrast (values normalized to the global maximum of the two plotted lines). Scale bars: 50 *µ*m.

To generate the two interfering sheets, we display two opposite sawtooth patterns simultaneously on the top and bottom halves of the SLM, as in Fig. 2a. As the beam diffracts off the SLM it is thereby split into two half-beams, propagating in the image plane (*xy* plane) but at two opposite angles *α* and −*α* from the optical axis of the launching arm. When the two half-beams meet again and interfere in the sample plane, they generate the desired sinusoidal pattern (Fig. 2b). The period of the final illumination pattern is defined by the propagation angle, which is in turn determined by the sawtooth period on the SLM. In order to shift the illumination pattern by a desired phase (to perform the 3-phase method), the equivalent optical phase shift can simply be added to one of the two halves of the SLM. Figure 3

shows results from the application of this structured illumination technique to image the heart of a formalin-fixed Zebrafish embryo specimen (4 days post-fertilization) expressing GFP fluorescence.

To quantify the improvement in image contrast obtained as a result of the out-of-focus background reduction, we calculated the standard deviation of the energy-normalized histograms [28] of the structured illumination (Fig. 3c) and the normal light-sheet images (Fig. 3b). This standard deviation can be calculated using the following formula:

$$\sigma_{N}\sqrt{\frac{\sum_{i}{\left(\frac{I_{i}-\bar{I}}{\sum I_{i}}\right)}^{2}}{C-1}},$$ (2)

where *C* is the total number of pixels in the image, *i* ranges from 1 to *C*, I*_i_* is the intensity value of the *i*-th pixel, ΣI*_i_* is the sum of all the pixels values in the image, and $\bar{I}=\sum I_{i}/C$ is the mean intensity value of the image. As more extensively explained in [28], the ratio between the *σ*_N_ values of two images can be used to quantify the change in image contrast, with an higher *σ*_N_ value corresponding to a better image contrast. For the images shown in Fig. 3b and 3c we calculated this ratio to be *σ*_N_(*c*)/*σ*_N_(*b*) ~ 2.6.

It should be noted that our approach to generate the two interfering light-sheets cannot be used with setup 2 and 3. In fact, in the case of perfect conjugation between the SLM and the waist of the light-sheet (or almost perfect conjugation with setup 3) the desired interference appears only on one side of the imaging FoV. Instead, the mis-conjugation offered by the side view of setup 1 moves the edge of the interference region to the side, allowing the two half-beams to interfere across the whole FoV. A possible alternative is to follow an approach analogous to the one used in [29]. The SLM would be divided into vertical stripes instead of into two halves, and the two blazed gratings displayed on alternate stripes. This method allows structured illumination experiments to be performed with all our three setups, but it gives rise to extra diffraction orders which reduce efficiency and, if not properly masked, increase out-of-focus excitation and bleaching. Masking these extra orders is possible, but we found that for our specific system it requires a careful selection of stripe size and design and alignment of the mask, complications which can be avoided when following our suggested approach.

### 3.2. Tiling

**Fig. 4:**
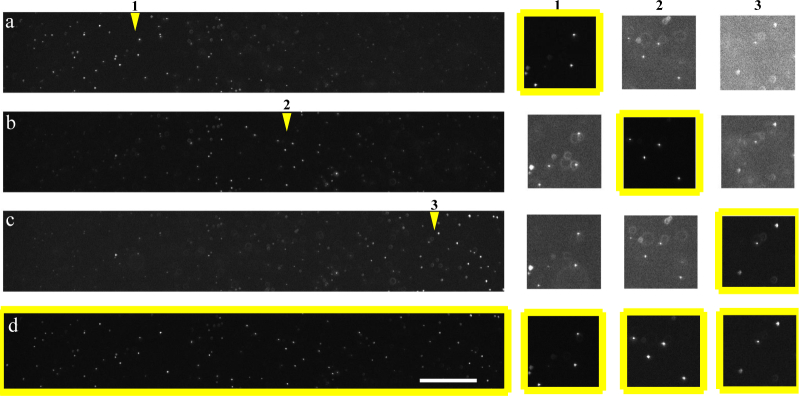
Tiling technique demonstrated with 0.2 *µ*m fluorescent beads. We acquired eight images of the same plane of beads, each image with the light-sheet focused at a different position (laterally) in the FoV. Left: (a,b,c) Same horizontal stripe taken from the second, fifth and eighth of the eight images taken (only three images shown for sake of clarity): in each image the position of the sheet waist (indicated by the yellow arrowheads) can be recognized by the brightness of the beads and the reduced number of out-of-focus beads. (d) Image obtained tiling the eight images, i.e. retaining only the sharpest vertical stripe taken from each of them. Right: zooms on beads taken from three different lateral positions (1, 2, 3) in the images, with each row showing how the same beads appear in the corresponding image to the left. Each of these zoomed images has been normalized to its own maximum value for clarity. Notice how, looking at one column at a time (i.e. the same sets of beads), as the position of the sheet’s waist gets further away from the position of the beads, the relative amount of light illuminating out-of-focus features increases. The composite tiled image (d) gives the best optical sectioning (most of the light concentrated on in-focus features) throughout the entire FoV. Scale bars: 50 *µ*m.

Tiling is a technique proposed to work around the trade-off between light-sheet thickness and length [13, 30]. The ideal light-sheet would stay thin across the whole imaging FoV. Light-sheets created by a focused Gaussian beam only remain thin over the beam’s Rayleigh length, and thus thinner sheets diffract and spread more rapidly as they propagate. To mimic the illumination delivered by an ideal thin and long light-sheet, one can use the waist of a short, thin sheet to image only one part of the FoV, then move the sheet’s waist laterally and build up, step by step, an image of the entire FoV. In our case, the light-sheet can be focused at different distances from the launching objective by adding a quadratic phase function (defocus) along the horizontal axis of the SLM. With such patterns, the light-sheet can be focused at different *x* positions in the imaging coordinate system. Images acquired by illuminating the same plane inside the sample but moving the focus of the light-sheet across the imaging FoV are then combined to obtain the final image. Of each of the initial images, only a restricted vertical stripe generated by the thin part of the light-sheet is allowed to contribute to the final image. Results obtained using this technique imaging fluorescent beads can be seen in Fig. 4

. For the tiling experiments we chose to use setup 1, which is the one that gives the thinnest light-sheet (best *z* resolution, but smallest effective FoV in *x*).

### 3.3. Multi-angle illumination for shadow suppression

To be imaged using SPIM, a sample first of all needs to be transparent enough for the light-sheet to propagate through it. Parts of the sample that strongly scatter or absorb the excitation light create a visible shadow behind them in the “downstream” direction of beam propagation (in our case to their right, since the light-sheet illuminates the sample from the left). This shadow effect can be reduced by combining illumination generated by light-sheets propagating at different angles [4, 5]. In both these previous works the illumination angle was modulated at high speed (> 1 kHz) to provide a range of illumination angles within a single image exposure. In our system, light-sheets with different propagation directions can be created by displaying different vertical sawtooth patterns (phase ramps) on the SLM. A shadow-free image can be obtained by switching between the different light-sheets within the exposure time of a single image, or by recording one image for each light-sheet inclination and combining the different images afterwards. This second approach, despite being less efficient in terms of image acquisition/computing time, offers the flexibility to permit what we propose as an alternative and improved algorithm for combining the acquired images to obtain shadow suppression: Maximum Intensity Projection (MIP).

**Fig. 5:**
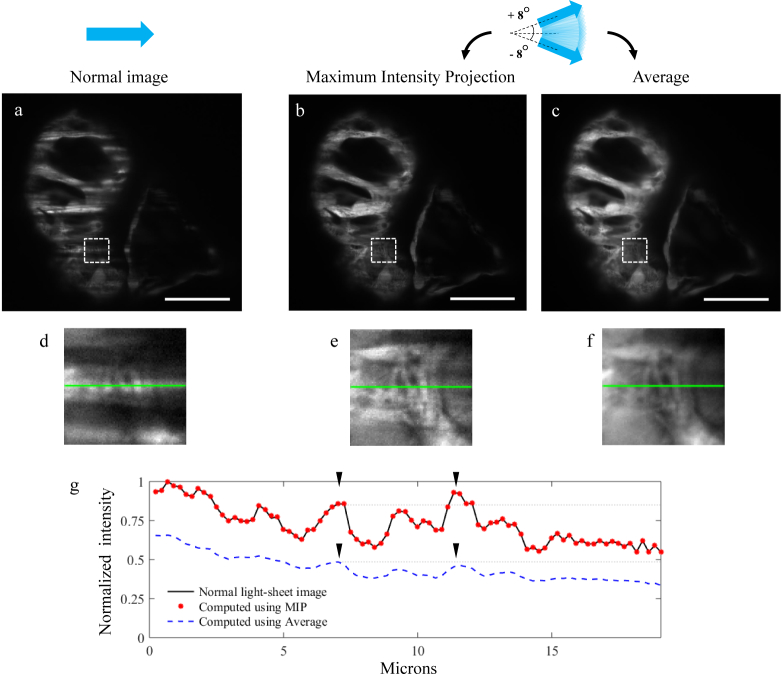
Shadow suppression using the light-sheet pivoting technique. (a) Image of a formalin-preserved Zebrafish embryo heart (4 dpf) acquired with a normal light-sheet, using setup 3. (b) Image obtained by computing the Maximum Intensity Projection (MIP) of a stack of seventeen images, acquired with the light-sheet propagating at different angles, equally spaced within ±8 degrees. (c) Image obtained by averaging the same seventeen images used for (b). (d–f) Zoomed-in views of the dashed line boxes in images (a–c). Each of these images has been normalized to its own maximum value. (g) Intensity profile of the same horizontal line in images (d–f). In order to obtain a reliable ground truth for comparison, in this specific case we selected a horizontal line that was already well-illuminated (not affected by shadows) in the normal light-sheet image (d), to which the profiles taken from (e) and (f) could be compared. This plot shows how the MIP allows to preserve the original image contrast and, with respect to averaging, a more accurate representation of the true intensity profile: notice how averaging (blue dashed line) distorts the relative intensity of the two peaks indicated by the black arrowheads, making the left peak appear as brighter than the one on the right. Intensity values in these plots are normalized to the global maximum of the three plotted lines. Scale bars: 50 *µ*m.

When the light-sheet is tilted through a range of different angles within a single image exposure, the resulting shadow-free image is generated from the *sum* of all the fluorescence excited by each light-sheet inclination. This technique can be replicated by computing the sum of a set of images acquired each with the light-sheet propagating at a different inclination. An alternative way of combining these images is to compute the Maximum Intensity Projection (MIP) of the whole stack: for each pixel, compare the values assigned to that pixel on each image and only keep the maximum one. This results in a final image where each pixel takes on the value from the raw image where that region was experiencing minimal shadowing. As can be seen in Fig. 5

, the image obtained using MIP not only preserves a better image contrast when compared to the one obtained by averaging, but it also assures a more accurate representation of the true intensity profile across the image. In fact, computing the average of a set of images acquired with the light-sheet propagating at different angles results in an alteration of the true image intensity profile: parts of the sample which are well-illuminated in all the images (i.e. are not affected by shadows) are inherently seen as brighter than those that are only illuminated in some of the images. Using MIP on the other hand ensures that the final intensity of each part of the sample only depends on the intensity observed when that part is illuminated without obstruction, and not on the number of images which *agree* with that intensity value. In Fig. 5 we compare a normal light-sheet image and the two alternative shadow suppression algorithms, averaging and MIP. A zoom-in on a region strongly affected by shadows shows the shadow suppression results obtained by combining seventeen images acquired with the light-sheet propagating at different angles, equally spaced within ±8 degrees. Before being combined either with MIP or averaging, the seventeen images were properly rescaled to account for the diffraction efficiency of the SLM, which changes with the angle of propagation of the diffracted first order, with a higher tilt corresponding to a dimmer light-sheet. We selected an horizontal line across a region of the sample which is not affected by any shadows in the normal light-sheet image (green line in Fig. 5(d)), and compared its intensity profile with the intensity profile of this same line in the two images computed for shadow suppression. This plot helps visualise the reduced image contrast offered by the averaging technique, and also its unpredictable distortion of the original intensity profile.

As mentioned in Section 2.1, these shadow suppression experiments on ex-vivo Zebrafish embryo heart are a good example of a situation in which the setup choice is strongly influenced by the type of sample. In fact, for the specific case of suppressing shadows in the Zebrafish heart we decided to use setup 3 instead of setup 2, which would normally be the one we would recommend using for shadow suppression experiments on samples with finer shadows. Let us discuss the reasons behind this in more detail:

The conjugation of the SLM plane with the center of the FoV is what makes setup 2 the most appropriate setup to be used for shadow suppression experiments, provided it can deliver a high enough tilt angle with high diffraction efficiency. As the SLM is used to send the first diffraction order to different directions, it generates light-sheets that propagate at different angles but overlap entirely again on each plane conjugate to the plane of the SLM, as they do on the SLM itself. In the case of setup 2, as the light-sheet is tilted using the SLM, its rotation in the sample plane happens around the center of the FoV, which is in fact conjugated to the plane of SLM. In setup 1 the situation is different: the rotation of the light-sheet happens around a position that is to the side of the FoV, such that a tilt also corresponds to an undesired vertical shift of the light-sheet in the images. As the tilt angle increases, the bright central part of the Gaussian sheet shifts away (vertically) from the FoV, which is illuminated by less and less light. There is however one other thing to consider: for shadow suppression the best results are obtained by using a large range of angles for the incoming light-sheets, particularly if thick shadows are present. In our system, a limit to the maximum achievable tilt angle is set by the SLM pixel spacing. By considering the magnification of the relay optics within our microscope, we can find the relation between the tilt angle of the light-sheet in the sample plane and the angle at which it originally propagates as it is diffracted off the SLM:

$$\sin(\theta_{2})=\frac{f_{1}f_{3}}{f_{2}f_{4}}\;\sin(\theta ),$$ (3)

where *θ* is the propagation angle after the SLM, *θ*_2_ is the propagation angle in the sample plane, and *f*_1_, *f*_2_, *f*_3_ and *f*_4_ are the focal lengths of the four lenses through which the beam passes before reaching the sample plane (see Fig. 1b), with *f*_4_ being the focal length associated with the launch objective. Finally, we can approximate sin(*θ*) with *λ*/*p* and obtain:

$$\sin(\theta_{2})=\frac{f_{1}f_{3}}{f_{2}f_{4}}\;\frac{\lambda }{p},$$ (4)

where *λ* is the wavelength (in our case 488 nm) and *p* is the pixel period of the sawtooth pattern displayed on the SLM: the smaller the period of the sawtooth pattern on the SLM, the larger the angle at which the light-sheet generated by the first diffracted order propagates. On the other hand, decreasing the sawtooth pattern period size also reduces the number of SLM pixels used for each period, and this coarser approximation of the ideal pattern results in a less efficient concentration of the diffracted light in the first diffracted order, i.e. the resultant light-sheet is dimmer. Even for larger pixel periods, this varying diffraction efficiency means that larger-angle sheets will be somewhat dimmer, and prior to further processing we rescale each image to compensate for the different brightnesses of the light-sheets they have been generated with.

In order to maintain a sufficiently bright first order, we required a minimum period of four SLM pixels. Using Equation 4 we can verify that a sawtooth period of four SLM pixels corresponds, with setup 2, to a tilt angle of ∼ 3°. For our experiments on the embryonic Zebrafish heart, we found that this maximum tilt angle was insufficient for good shadow suppression. We therefore decided to perform these experiments with setup 3, which gives an adequate enough (even though not perfect) conjugation between the SLM plane and the center of the imaging FoV but offers a much bigger range of possible tilt angles, with a 50 *µ*m period (four SLM pixels) corresponding to a tilt angle of ∼ 8.8°. Figure 5 illustrates the results obtained combining seventeen images acquired with setup 3, with the light-sheet propagating at different angles, equally spaced within ±8°, imaging the heart of an ex-vivo 4 dpf Zebrafish embryo.

### 3.4. Pencil beam scanning (synthetic DSLM)

Part of the fluorescence excited in the illuminated plane undergoes scattering by the intervening tissue before reaching the detector, resulting in an increased diffused background signal and a loss of contrast. In DSLM light-sheet systems, where the light-sheet is formed by a rapidly-scanned 2D Gaussian beam (a “pencil” beam), the beam scan can be combined with a confocal rolling shutter on the camera to suppress the background signal [10]. We can replicate this same approach by turning our light-sheet into a pencil beam using the SLM: we can record a sequence of full-frame images as the pencil beam is scanned across the FoV (still using the SLM), and then create the final image by applying a synthetic confocal slit to each raw image (i.e. masking out all rows except those where the pencil beam should have appeared). This post-acquisition confocal slit method is implemented for example in [7], where each image is multiplied by a smooth Gaussian mask centered and aligned with the illumination beam. In order for the mask to correctly select the desired part of each image, the position and inclination of the illumination beam needs to be known, either from previous calibration or from the acquired images. In fact, both implementations of the confocal slit technique (rolling shutter, and post-acquisition masking) require precise alignment and size-matching between the illumination beam and the detection line used.

Our system enables us to implement the post-acquisition confocal slit method, and we here propose an alternative way to process the raw images and achieve background rejection, obtaining a technique that combines the ease of a standard, full-frame acquisition with a simple and calibration-free postprocessing procedure. We scan the illumination beam across the entire FoV, acquiring a single full image for each position of the beam. We then combine these images into a 3D stack of size *n* × *m* × *i*, where *n* × *m* is the image size in pixels and *i* is the number of images taken. The final contrast-enhanced image can be obtained by simply computing the Maximum Intensity Projection of the stack of images along its third dimension (dimension of size *i*).

To explain and justify this procedure, let us concentrate on how the intensity value of a single pixel changes as we scan through the stack of images. Let (*x*, *y*) be the position of the pixel in the image, and (*x_s_,y_s_*) its corresponding location in the sample plane, and assume we expect to detect some non-scattered signal from (*x_s_,y_s_*). The value of pixel (*x,y*) will be very low in the images taken with the pencil beam positioned far away from (*x_s_,y_s_*), somewhat higher as the beam gets closer to it and more scattered light reaches pixel (*x*, *y*), and it will be at its highest when (*x_s_,y_s_*) is directly in the path of the incoming beam. The Maximum Intensity Projection therefore gives, for each pixel (*x*, *y*), the intensity received by it in the image recorded with the illumination beam giving best overlap with (*x_s_,y_s_*), the position in the sample that maps onto that pixel. And this is exactly what we wish to retain in our reconstructed image.

Acquiring entire images but then only retaining some pixel rows from each image is of course a relatively slow and inefficient way of implementing the pencil beam scanning technique. However, this shows how our flexible imaging system can easily be used to explore the feasibility of new imaging modalities, obtaining good pilot results without investing the time and effort needed to build and calibrate a high-speed dedicated rolling-confocal-slit DSLM system. Our post-processing approach based on a simple Maximum Intensity Projection also has the benefit of not requiring any calibration of the pencil beam position and orientation on each image plane.

In our implementation the sample is illuminated by a regular 2D-focused beam, which we generate by displaying an opposing cylindrical lens phase function on the SLM. This corresponds to adding a quadratic phase function (defocus) along the vertical axis of the SLM, to undo the effects of the physical cylindrical lens (note that the same could be achieved by physical removal of the cylindrical lens from the system, combined with flat SLM). The result of this phase modulation is a beam which is focused in front of the launching objective along both *y* and *z*. The SLM is also used to scan the beam and sequentially illuminate, line by line, the entire in-focus plane. The phase function applied to our SLM is therefore the sum of the cylindrical lens pattern (to generate a focused beam) and a linear phase ramp (to vertically move the focus of the beam). In practice the combination of these two functions yields a cylindrical phase function that translates vertically on the SLM as the pencil beam is scanned (or laterally on the SLM, in case of our experiments with the glycerol setup).

**Fig. 6:**
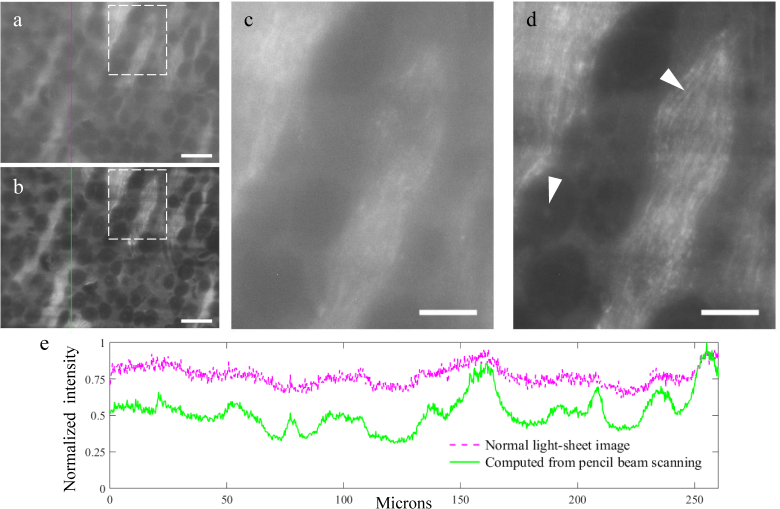
Pencil beam scanning technique applied on a cleared whole mouse brain sample, imaged in glycerol. (a) Image acquired with a normal light-sheet. (b) Image generated with the scanning pencil beam technique, using our reconstruction procedure (based on a Maximum Intensity Projection) on a set of 200 raw images, each taken with the horizontal pencil beam focused at a different height in the sample plane. Scale bars: 50 *µ*m. (c,d) Zoomed-in views of the dashed line rectangles in images (a,b), to highlight some of the faint features (left arrowhead) and fine structures (right arrowhead) revealed by the pencil beam scanning technique. Scale bars are here of 20 *µ*m. (e) Cross section of the normalized intensity along the same column in images (a) and (b) (values normalized to the global maximum of the two plotted lines).

The improved contrast achievable using this technique is particularly valuable when imaging deep in highly scattering samples. To demonstrate this we performed the pencil beam scanning technique on cleared whole mouse brain samples, with results shown in Fig. 6

. For this experiments we programmed the SLM to make the pencil beam translate with steps of 1.4 *µ*m in the sample plane (corresponding to ∼6 pixels in the image, with the pencil beam having a FWHM of ∼ 25 pixels). A total of 200 images were taken to cover the region of interest shown in Fig. 6 (1146×1556 pixels).

### 3.5. Autofocusing

In our system (as with most light sheet microscopes) 3D imaging is performed by scanning the sample through the light-sheet using a motorized stage, which can be synchronized with the camera acquisitions. This means that, during the process of acquiring a stack of images at different depths in the sample, the relative position of the light-sheet with respect to the imaging objective does not change, and the initial focus is maintained. Nevertheless, it is very important, at the beginning of every imaging session, to check and eventually adjust the image focus. In the SLM-SPIM the light-sheet can be moved in *z*, i.e. towards and away from the imaging objective, by displaying a horizontal phase ramp on the SLM. This offers a natural method for optimizing the position of the light-sheet to coincide with the focal plane of the camera, without having to move the imaging objective or the tube lens. Particularly for high-numerical-aperture imaging in thick samples, this optimization must often be performed on a per-sample basis, and even when moving to a different location in the sample.

**Fig. 7:**
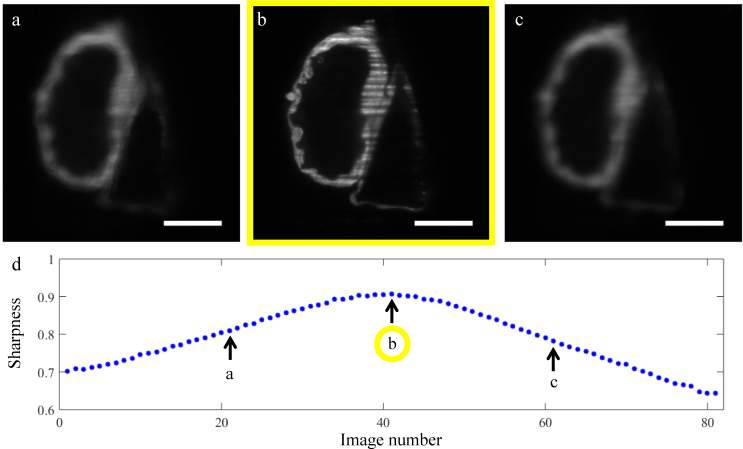
Autofocusing experiment using sharpness metric of Equation 5 on an ex-vivo 4 dpf Zebrafish embryo’s heart (using setup 1). In this illustration, eighty-one images of the same plane inside the sample were taken using the SLM to move the sheet to different positions with respect to the imaging objective, and the motorized stage to move the sample with the light-sheet (with steps of 0.5 *µ*m, for a total range of 40 *µ*m). (a) and (c) show images taken with the light-sheet in an out-of-focus plane, while (b) is the image identified as the one with best focus according to the sharpness metric (i.e. light-sheet at the correct distance from the imaging objective). (d) Sharpness values, one for each image, with highest value indicating the plane of best focus. Note that in practice a smaller number of images would be taken, and the sharpness interpolated using an appropriate function, to quickly find the optimum focus. Scale bars: 50 *µ*m.

We developed a MATLAB script to automatically optimize the light-sheet position to the plane of best focus. First, the light-sheet is scanned over a certain range in *z* (chosen by the user) around its rest position (flat SLM), recording one image for each position of the light-sheet. During this scan, the sample is moved together with the light-sheet using the motorized stage, so that the relative position of the light-sheet with respect to the sample is fixed (i.e. the same plane inside the sample is imaged at each step, to ensure any change in contrast is purely due to the change in defocus of the *same* sample plane). The images are then analyzed, and the light-sheet (together with the sample) is moved to the position that yielded the image with best focus. To evaluate the quality of the focus of each image, we use the sharpness metric proposed in [31] and used for adaptive optics on a SPIM in [32]. This metric quantifies the image focus through a measure of the ratio between the high and low spatial frequency content of the image, and is defined as follows:

$$S=\frac{\sum_{N_{p}}{\left|\;ℱ[I(x,y)]\;\right|}_{\text{masked}}}{\sum_{N_{p}}{\left|\;ℱ[I(x,y)]\;\right|}_{\text{unmasked}}},$$ (5)

where I(*x, y*) is the intensity of pixel (*x*, *y*), N_p_ is the number of pixels in the image, and ℱ denotes the Fourier transform. A rectangular mask is applied to the 2D power spectral density (PSD) of the original image (ℱ[I(*x, y*)]), in order to mask out its central values, representing the lowest spatial frequencies contained in the image. The sharpness value S is then given by the sum of the absolute values of ℱ[I]_masked_ divided by sum of the absolute values of ℱ[I]_unmasked_ (0 < S < 1). As the images become more blurred (moving away from the plane of best focus), their low spatial frequency content increases with respect to the high frequency content, which means that the mask that subtracts the lowest frequencies has a stronger effect on ℱ[I], resulting in a lower value for S. The maximum S value identifies the image with best focus (see sharpness plot in Fig. 7

). In the experiment presented in Fig. 7 the sharpness metric was calculated using a band-pass mask with a cut-on spatial frequency (in the sample plane) of 0.009 *μ*m^−1^(11×9 pixels) and a cut-off spatial frequency of 0.2275 *μ*m^−1^ (201×151 pixels).

## 4. Conclusion

We have shown how a phase-only liquid crystal SLM can be introduced as a simple modification to the illumination arm of a SPIM, to give a flexible, versatile system able to deliver high quality images by applying a range of advanced light-sheet imaging techniques. Imaging fluorescent beads, Zebrafish embryos and optically cleared whole mouse brain samples, we have demonstrated how the SLM-SPIM can be used to apply: structured illumination and pencil beam scanning techniques to reduce the out-of-focus content of the images; light-sheet pivoting to reduce the effect of shadows; light-sheet tiling to obtain a more uniform illumination across the image FoV and improve optical sectioning; automated focus optimization. Our modular system also gives the option to choose between three different light-sheets, allowing to select the sheet’s thickness and height according to the characteristic of the sample and the imaging technique to be performed. We have also proposed new, computationally-undemanding image reconstruction methods based on the maximum intensity projection operation.

With its simple, functional design and the use of a computer-reconfigurable SLM, we believe our system represents an ideal platform for manipulating the illuminating light-sheet to apply a range of advanced imaging techniques on a single SPIM microscope, and also to explore combinations of multiple techniques and potentially trial new ones.
